# The Retinoblastoma Tumor Suppressor Regulates a Xenobiotic Detoxification Pathway

**DOI:** 10.1371/journal.pone.0026019

**Published:** 2011-10-12

**Authors:** Maria Teresa Sáenz Robles, Ashley Case, Jean-Leon Chong, Gustavo Leone, James M. Pipas

**Affiliations:** 1 Department of Biological Sciences, University of Pittsburgh, Pittsburgh, Pennsylvania, United States of America; 2 Department of Molecular Virology, Immunology and Medical Genetics, College of Medicine, The Ohio State University, Columbus, Ohio, United States of America; 3 Department of Molecular Genetics, College of Biological Sciences and Comprehensive Cancer Center, The Ohio State University, Columbus, Ohio, United States of America; Institut Jacques Monod, France

## Abstract

The retinoblastoma tumor suppressor (pRb) regulates cell cycle entry, progression and exit by controlling the activity of the E2F-family of transcription factors. During cell cycle exit pRb acts as a transcriptional repressor by associating with E2F proteins and thereby inhibiting their ability to stimulate the expression of genes required for S phase. Indeed, many tumors harbor mutations in the RB gene and the pRb-E2F pathway is compromised in nearly all types of cancers. In this report we show that both pRb and its interacting partners, the transcriptional factors E2F1-2-3, act as positive modulators of detoxification pathways important for metabolizing and clearing xenobiotics—such as toxins and drugs—from the body. Using a combination of conventional molecular biology techniques and microarray analysis of specific cell populations, we have analyzed the detoxification pathway in murine samples in the presence or absence of pRb and/or E2F1-2-3. In this report, we show that both pRb and E2F1-2-3 act as positive modulators of detoxification pathways in mice, challenging the conventional view of E2F1-2-3 as transcriptional repressors negatively regulated by pRb. These results suggest that mutations altering the pRb-E2F axis may have consequences beyond loss of cell cycle control by altering the ability of tissues to remove toxins and to properly metabolize anticancer drugs, and might help to understand the formation and progression rates of different types of cancer, as well as to better design appropriate therapies based on the particular genetic composition of the tumors.

## Introduction

Organisms respond to xenobiotics -natural compounds or artificial substances not normally present in the body such as drugs, antibiotics, pollutants and carcinogens- by deactivating and excreting those products via a series of enzymes located mostly in the liver and, to a lesser extent, in the small intestine. Three sets of enzymes contribute to the process. First, Phase I enzymes chemically modify the xenobiotics by multiple mechanisms. Phase II components then conjugate the products with glucuronic acid, sulphuric acid or glutathione, to make them more soluble. Finally, transporter members of the Phase III help to excrete the modified components via urine or bile [1]. Phase I of the pathway is carried out by members of the cytochrome P450 (Cyp) superfamily, a large and diverse group of hemoproteins present in most organisms and whose activity is responsible for almost 75% of the total drug metabolism in higher eukaryotes [1]. Phase II involves the conjugation of modified xenobiotics by transferases like glutathione s-transferases (GSTs) and UDP-glucuronosyl transferases, which normally results in less active metabolites that are also more soluble in water [1]. Drug transporters such as the ATP-binding-cassette (ABC) superfamily comprise Phase III of the detoxification pathway. They eliminate and distribute the less active, more soluble products from Phase II metabolism [1]. The synthesis of several Cyp enzymes is induced in response to specific drugs (naptoflavone, PCN) or naturally occurring molecules (bergamottin, paradisin-A). In some cases the enzyme activity is also modified by interaction with the drug. As changes in Cyp activity will affect the metabolism and elimination of various drugs, understanding and identifying genetic factors that can modify the detoxification response is especially important when using drugs with noticeable side-effects, with small therapeutic windows or necessary to treat critically ill patients.

The product of the retinoblastoma gene (pRB) and its two related proteins, p107 and p130, control the transition between G1 and S phase, thus preventing abnormal cell proliferation. They function by interacting with the E2F family of transcription factors, which in turn regulate multiple genes essential to progress through the G1-S phase. Two groups of factors can be identified within the mammalian E2F family, according to their biochemical properties, effects of target genes, and expression through the cell cycle: E2F1-2-3 in one hand and E2F4 through E2F8 on the other (reviewed in [2],[3]. The work of many groups indicate that pRb proteins binding to E2F factors either prevents E2F-dependent transcriptional activation or indeed promotes active repression by recruiting chromatin remodeling complexes and histone modifying activities to the promoter, thus effectively blocking S-phase progression and suppressing undue cell proliferation. However, while E2F1, 2 and 3 were originally classified as transcriptional activators by *in vitro* assays, we have recently reported a role of E2F1, 2 and 3 and pRb in transcriptional repression, cell cycle exit and cell survival [4].

The association between pRb proteins and E2Fs is normally regulated by cyclin-dependent phosphorylation. However, exogenous factors such as viral oncoproteins are able to disrupt the pRb/E2F complexes, thus their expression results in uncontrolled proliferation and/or tumorigenesis. In particular, the large T antigen (TAg) encoded by Simian Virus 40 (SV40) has been shown to bind and inactivate members of the pRb pathway, resulting in upregulation of E2F proteins and E2F activity, with the subsequent induction of cellular proliferation and tumorigenesis in different systems, both *in vivo* and *in vitro* ([5], [6] [7]). TAg binds the pRb proteins through its LXCXE peptide motif, and mutations in TAg altering or eliminating the LXCXE domain fail to interact with the pRb proteins and to induce cell proliferation. In addition to its role inducing cell proliferation and tumorigenesis, we have previously shown that, when expressed ectopically in intestinal enterocytes, TAg also downregulates the endogenous and constitutive RNA levels of multiple detoxification components from Phases I, II and III [8]. This effect requires an intact LXCXE motif in TAg, suggesting that perhaps the transcription of detoxification components normally requires pRb proteins. We have explored this hypothesis and confirmed that pRb is required to achieve normal levels of detoxification enzymes and, furthermore, that depletion of activator E2Fs (E2F1-2-3) seems to play a similar role. Our results are indicative that both pRb and E2F1-2-3 act as activators of the detoxification system and have important implications for understanding tumor progression and tumor treatment.

## Results and Discussion

Organisms respond to xenobiotics by deactivating and excreting those products via a series of enzymes, which, in mammals, are located mostly in the liver and small intestine. Three sets of enzymes contribute to the process: Phase I components chemically modify xenobiotics by the cytochrome P450 (Cyp) superfamily, whose activity is responsible for almost 75% of the total drug metabolism. Subsequently, Phase II components, like glutathione s-transferases (GSTs) and UDP-glucuronosyl transferase (UGTs), conjugate the modified xenobiotics to render less active metabolites that are also more soluble in water. Finally, transporter members of the ATP-binding-cassette (ABC, Phase III) help to excrete the modified components via urine or bile [9], [10], [11], [12],[13].

The E2F factors regulate multiple genes essential to progress through S phase, and binding of E2Fs by the retinoblastoma (pRb) related proteins effectively blocks S-phase progression and suppresses cell proliferation [3], [14], [15]. Associations between pRb proteins and E2Fs are normally regulated by cyclin-dependent phosphorylation, ensuring a smooth transition through the cell cycle. Exogenous factors, including viral oncoproteins like SV40 T antigen (TAg), can disrupt the pRb/E2F complexes, resulting in upregulation of E2F target genes, uncontrolled proliferation and/or tumorigenesis both *in vivo* and *in vitro* [16], [17], [7]. An LXCXE peptide motif in TAg is necessary to bind the pRb proteins and to induce cell proliferation.

**Figure 1:**
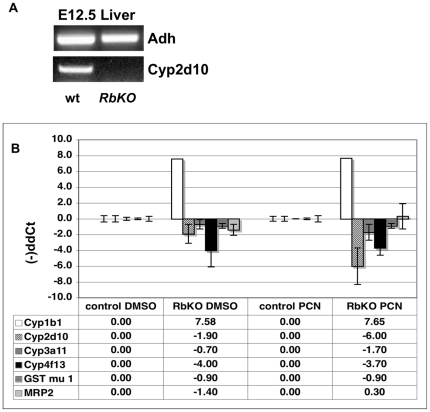
Impaired detoxification response in livers lacking pRB in the presence or absence of chemical challenge. Conventional RB +/- females pregnant from RB +/- males were injected with PCN or DMSO -as control- 24 hours prior to sacrifice. RNAs were obtained from livers of embryos at the indicated times and the corresponding cDNAs were analyzed. a) Unlike the control counterparts, E12.5 RBKO embryonic livers did not show induction of Cyp2d10 by RTPCR upon PCN treatment. Adh was used as normalizing control and samples were repeated in triplicate. RB +/- embryos showed similar Cyp2d10 levels to those on normal controls. b) Real Time PCR analysis was used to determine the levels of Phase I, Phase II and Phase III components in E18.5 livers. Downregulation of most detoxification genes (Phases I, II and III) was observed both, with or without PCN induction in RBKO background.

In addition to the induction of ectopic proliferation, expression of TAg results in the mRNA downregulation of detoxification components from Phases I, II and III in intestinal enterocytes [8]. This effect requires an intact LXCXE motif in TAg, suggesting that pRb might control the transcription of detoxification components. To explore this possibility, we examined the RNA levels of different members of Phases I, II and III in genetic background devoid of pRb. Conventional inactivation of pRb in mice results in numerous alterations and null embryos die around E13.5 [18],[19], [20], hampering the study of possible pRb effects prior to birth. Nevertheless, pRb-deficient embryos supplied with a wild-type placenta are viable, but die soon after birth [21]. Using such mice allowed us to analyze the effects of pRb ablation in tissues from embryos and newborn mice. At E12.5, the earliest point analyzed, the endogenous expression of detoxification components is absent or barely detectable even by *in situ* hybridization (http://www.genepaint.org). However, the transcription levels of Cyp2d10 in livers of control embryos generated from females treated with PCN (pregnanolone-16 alpha-carbonitrile) an inducer of the detoxification pathway, are substantially increased, while are not detectable in *RBKO* littermates (Figure 1a). Furthermore, removal of pRb in E18.5 liver samples, at which point endogenous and inducible levels of several detoxification components are detectable, results in overall reduction of both endogenous and PCN-induced levels of Phases I, II and III transcripts, with the exception of Cyp1b1 (Figure 1b).

**Figure 2:**
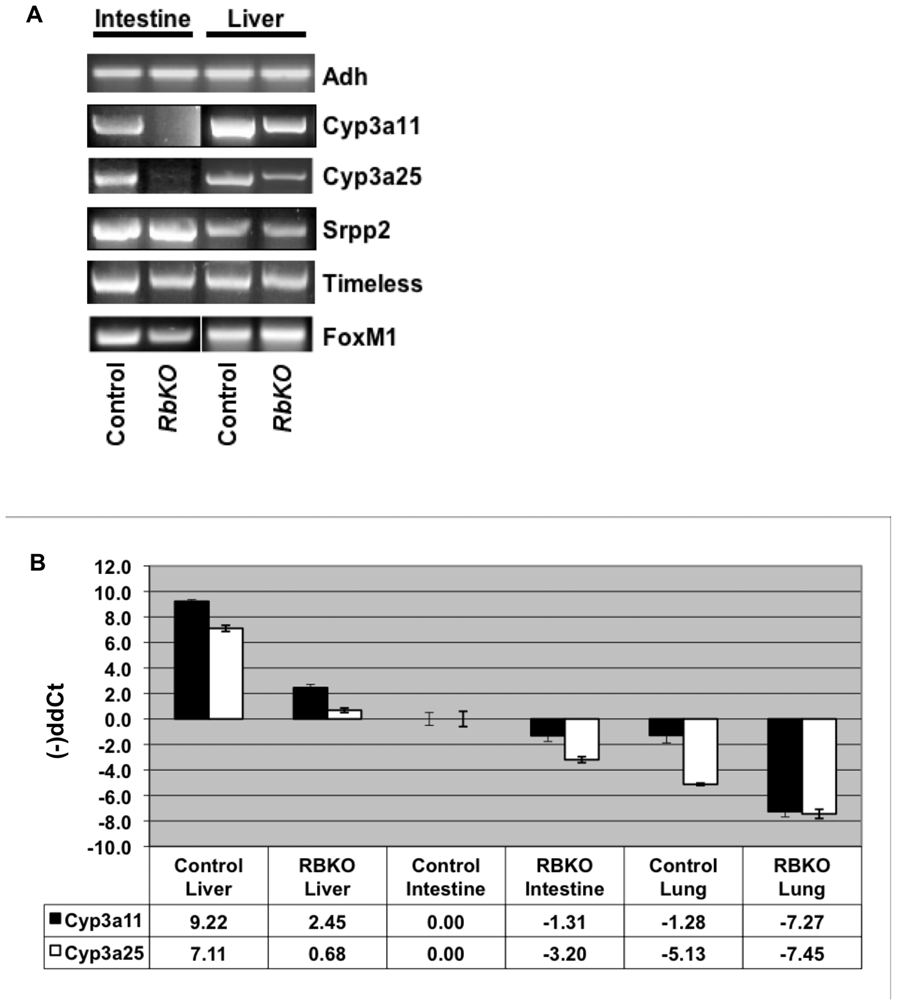
Phase I mRNA components are significantly reduced in several newborn tissues in the absence of pRb. a) RNAs from intestines or livers of newborn mice were converted to cDNAs and analyzed by RTPCR with probes specific for different genes. A reduction of Phase I transcripts was observed in RBKO samples in contrast with control samples, while no changes were observed in several other genes tested. Adh levels are used as endogenous controls. b) RNAs from newborn tissue samples were analyzed by Real time PCR, indicating that Cyp3A11 and Cyp3a25 mRNAs decrease substantially in samples lacking a functional RB gene in all tissues tested: liver; small intestine and lungs. All samples were normalized against Rpl5 and compared against those present in control small intestines.

We analyzed several tissues in newborn mice, and confirmed that removal of pRb results in a reduction of endogenous Cyp levels in newborn livers and intestines, while other transcripts used as control remain unaltered (Figure 2a). This effect was confirmed in all the tissues expressing Cyps at that specific developmental stage, including liver, intestine and lungs (Figure 2b). Other tissues analyzed from E18.5 to newborn mice, including brain, heart and spleen, did not show detectable RNA levels of members of the detoxification pathway with or without chemical challenge (data not shown). Thus, it appears that pRb is required for constitutive expression and induction of the Cyp pathway.

**Figure 3:**
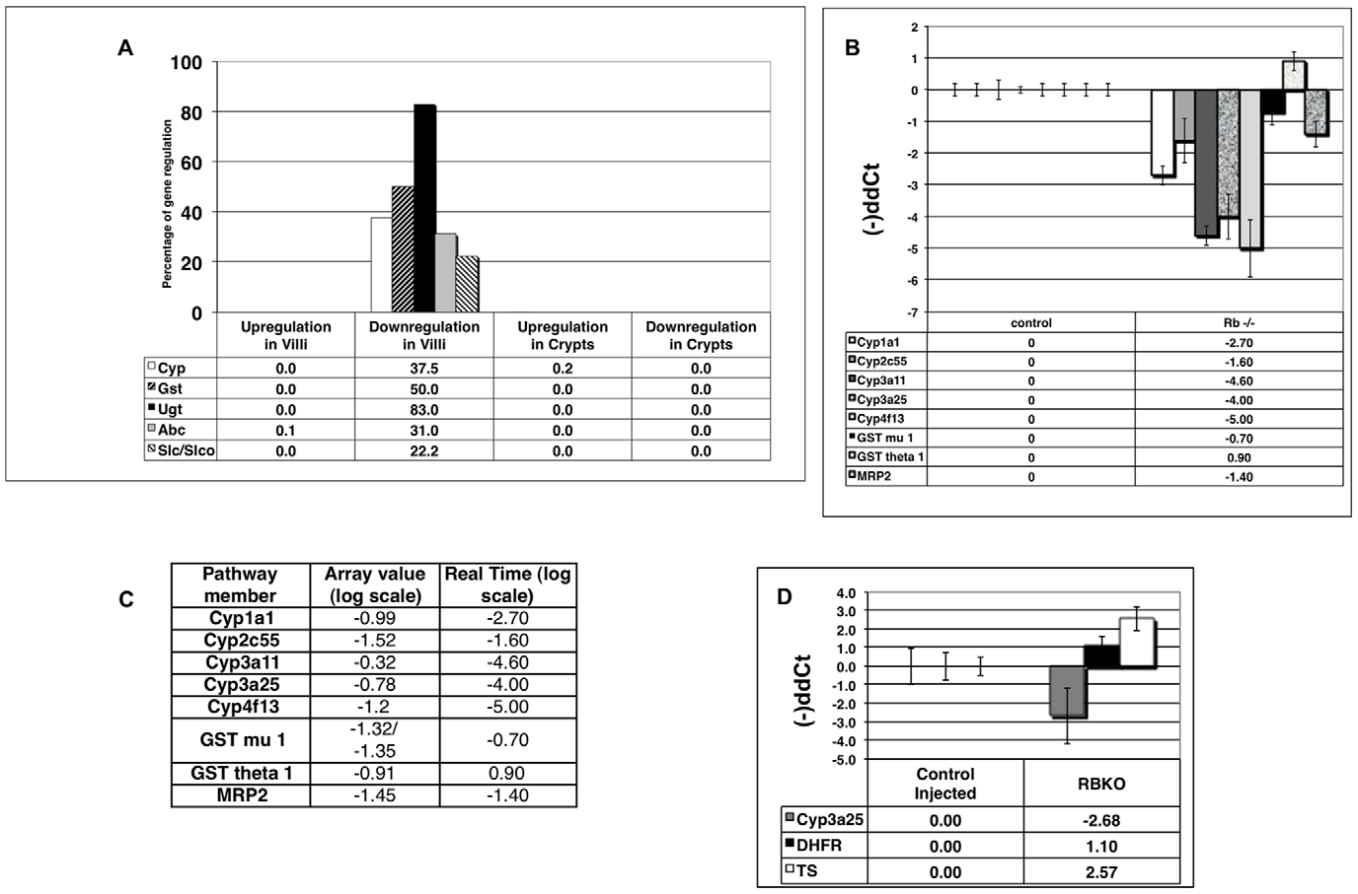
Transcriptional downregulation of members of the detoxification pathway in villi of RbKO mice. a) Microarray values obtained from RBKO villi were compared to corresponding ones from control littermates. The percentage of genes up- or downregulated 1.5 fold within each specific group is represented, indicating a clear downregulation of the detoxification genes in the absence of a functional pRb product. b) Confirmation of gene downregulation by Real Time PCR analysis. Adult villi RNAs from control and RBKO mice were subjected to Real time PCR analysis with primers for specific genes of the detoxification pathway. The results were normalized against endogenous controls (Adh and/or Rpl5). c) Comparison between levels of detoxification components in RBKO versus control villi as observed in microarray or Real Time PCR analysis. d) Downregulation of detoxifying components in villi lacking pRb is in contrast with the upregulation observed in E2F target genes (TS and DHFR). Real Time PCR analysis revealed that E2F target genes were increased in RBKO villi, while Cyp3a25 levels showed a drastic reduction. The values represent the average of three independent samples.

Next, we examined mice where *RB* had been conditionally deleted in the intestinal epithelium (*RBKO* mice; [4]). We have previously published the characterization and validation of the global gene expression profile in villi and crypts from these mice, as well as from mice lacking all three activator E2Fs *(E2F1-2-3 KO* (*TKO*), and mice lacking both a functional *RB* and *E2F1-2-3* (*QKO*) [4]). A summary of the detoxification genes analysis in *RBKO* background is shown in Figure 3a and Supplementary Table S1. Overall, the expression levels of 37.5% of Phase I genes, 57.7% of Phase II genes and 28% of Phase III genes are reduced in the absence of a functional RB gene. The expression levels of genes corresponding to Phases I, II and III of the detoxification pathway are significantly reduced in *RBKO* villi samples. This is highly significant, as, overall, only 875 genes (8.1%) were downregulated 1.5 fold in *RBKO* background, 827 genes (7.7%) were downregulated 1.5 fold in *TKO* villi, and 820 genes (7.6%) were downregulated 1.5 fold in *QKO*. The set of genes downregulated in all *RBKO*, *TKO* and *QKO* backgrounds in villi amounted to 156 (1.4%).

We used the default settings with the DAVID database (http://david.abcc.ncifcrf.gov) to annotate genes downregulated in each of the examined backgrounds. In *RBKO* background, the fourth cluster (enrichment score 4.87) is made of members of the detoxification pathway, with p values ranging from 1.7E-9 to 4.6E-2. In *TKO* background, the first cluster (enrichment score 5.57) is composed of members of the P450 pathway, drug metabolism and Gst proteins, with p values ranging from 3.9E-13 to 3.8E-2. In *QKO* background, the 2nd cluster (enrichment score 4.38) is made of members of the P450 pathway, drug metabolism and Gst proteins, with p values from 3.4E-12 to 1.3E-2. Finally, the first cluster (enrichment score 5.06) of genes downregulated in all backgrounds is composed of members of the P450 pathway, drug metabolism and Gst proteins, with p values from 1.1E-12 to 2.2E-3.

**Figure 4:**
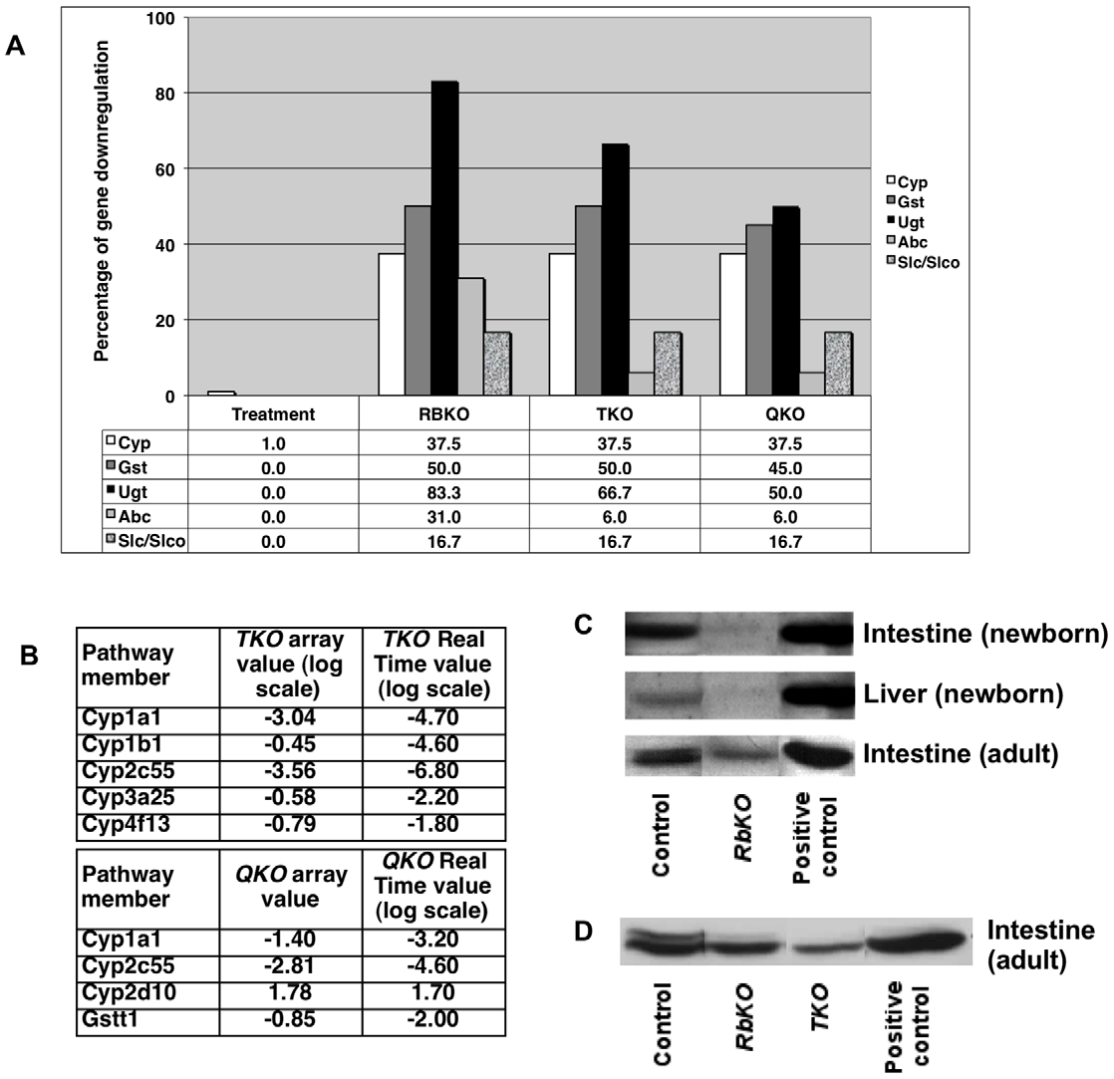
Downregulation of detoxification genes in villi lacking functional pRb and/or E2F1-2-3 proteins. a) Samples from villi or crypts of RBKO; TKO or QKO mice were compared to corresponding ones from control β-NF-injected mice. The percentage of genes downregulated 1.5 fold versus the control is represented within each specific group of detoxification genes, indicating a clear reduction of the detoxification pathway in the b) Confirmation of gene downregulation by Real Time PCR analysis of drug metabolizing enzymes in TKO and QKO villi samples in comparison to values obtained by microarray. Values represent the levels observed in TKO or QKO mice versus control mice. Samples were normalized against an endogenous control (Adh and/or Rpl5). c) Cyp3A protein levels decrease in samples lacking pRb. RIPA-extracted protein extracts were prepared from intestines or livers of newborn mice, or from adult fractionated intestines containing villi and crypts as indicated. Control RB +/+; experimental RB -/- and positive control samples -from adult mouse liver- are indicated. Following SDS-PAGE gels, western blot analysis with anti-Cyp3a antibody indicates a substantial decrease in Cyp3A levels in all samples lacking a functional pRb. d) Removal of either pRb or E2F1-2-3 results in reduced levels of Cyp3A protein in adult intestinal samples. RB +/+ (control); RB -/- (RbKO); E2F1 -/-; E2F2 -/-; E2F3 loxP/loxP (TKO) and positive control (adult liver) samples are indicated.

To confirm the reduced expression of detoxification genes in *RBKO* background, we analyzed RNA villi samples by Real Time PCR analysis (Figure 3b). With the exception of Gst theta 1, these experiments confirm that the absence of pRb results in downregulation of almost all detoxification genes tested and, indeed, values obtained by microarray analysis correlate very well with those obtained by Real Time PCR (Figure 3c). In contrast to the Cyp pathway, canonical E2F target genes were strongly upregulated in villi of *RBKO* mice (Figure 3d) and [4].

Many of the effects attributed to pRb in controlling the cell cycle are mediated through its interaction with E2F transcription factors. In the classic view of cell cycle control, pRb and activator E2Fs (E2F1-2-3) play antagonistic roles, and thus we examined the possible role of E2F1, 2 and 3 in regulating the levels of detoxification genes. Microarrays comparing intestinal villi from mice lacking functional *E2F1, E2F2* and *E2F3* genes in the intestinal epithelium (triple KO or *TKO* mice) and control littermates [4] were examined for effects on the detoxification pathways. We found that the expression of genes from Phases I and II of the detoxification pathway is significantly reduced in villi of *TKO* mice, while Phase III is only marginally affected. Overall, the expression levels of 37.5% of Phase I genes, 53.8% of Phase II genes and only 8% of Phase III genes were reduced in the *TKO* villi (Figure 4a and Supplementary Table S1). These results suggest that pRb and E2F1-2-3 do not function antagonistically, but rather are likely components of a common mechanism impinging on the control of drug metabolizers.

These results were somewhat surprising as, in the classical view of cell cycle control, pRb and E2Fs play antagonistic roles and pRb has been shown to negative regulate E2F activity. However, we have recently reported a coordinated role of pRb and E2F1-2-3 in transcriptional repression, cell cycle exit and cell survival [4]. Therefore we evaluated expression of detoxification genes in villi samples from mice lacking pRb as well as E2F1, 2 and 3 (*QKO* mice). The overall pattern mirrors the ones obtained with *RBKO* and *TKO* samples. The expression levels of 37.5% of Phase I genes, 46% of Phase II genes and 12% of Phase III genes were reduced in villi in the absence of RB and E2F1, 2 and 3 genes (Figure 3a and Supplementary Table S2). Real Time PCR analysis of specific genes in *TKO* and *QKO* villi (Figure 4b) reiterates that *TKO* and *QKO* samples show a significant reduction in the expression of detoxification genes, validating the microarray results (Supplementary Tables S2 and S3). These results indicate that E2F1-2-3 and pRb cooperate to positively control the transcriptional levels of detoxification genes. At this point we do not know if pRb/E2F complexes contribute directly or indirectly to control the detoxification pathway.

To determine if the observed changes in mRNA abundance are reflected in protein levels, we analyzed the levels of Cyp3A proteins, one of the most important subfamilies involved in the metabolism of xenobiotics, in intestinal and hepatic samples. All samples tested, including newborn intestines and livers as well as murine adult villi, showed a considerable decrease in Cyp3A proteins in the absence of pRb (Figure 4c). Furthermore, removal of either pRb or E2F1-2-3 leads to decreased Cyp3A protein levels in the intestinal epithelium of adult mice (Figure 4d). Thus, transcriptional downregulation of Cyp3A correlates with reduction in protein levels in intestinal and hepatic tissues.

Our results demonstrate that both pRb and one or more of the activator E2Fs (E2F1-3) are required for basal expression and xenobiotic induction of several members of the detoxification pathway. Active pRb controls cell proliferation by negatively regulating the activator E2Fs (E2F1-3). Thus, ablation of pRb leads to E2F-dependent gene transcription and cell proliferation. We have recently shown that both pRb and the activator E2Fs are also required to establish repression of E2F-dependent transcription as progenitor cells exit the cell cycle and differentiate [4]. Under these conditions other RB family members cannot compensate for pRb loss, suggesting that pRb-E2F1-3 complexes must first bind E2F-dependent promoters prior to the establishment of the permanent repressing complex, which consists of p130-E2F4. Furthermore, under some stress conditions such as DNA damage, pRb, and not p107 or p130, is involved in blocking the cell cycle. Perhaps tissues respond to chemo- or genotoxic stress by inducing pRb, which, in cooperation with the activator E2Fs, blocks cell proliferation and induces the detoxification response. This model suggests that the loss of pRb function during tumorigenesis may have effects on the ability of tumor cells to metabolize and eliminate toxins or to properly metabolize anticancer drugs. Specifically, our model would predict that compounds rendered less toxic by the detoxification pathway would be more genotoxic in *RBKO* cells. In fact, *RBKO* liver cells are more susceptible to tumorigenesis after treatment with aflatoxin B1, a drug converted to less toxic products by P450 enzymes [22]. Similarly, our model predicts that cancer treatment with drugs activated by the P450 pathway (such as tamoxifen) would not be as effective in *RB* null background. Consistent with this, *RB* deficiency is associated with recurrence of breast cancer following tamoxifen therapy (reviewed in [23]). Thus, the *RB* status impinges on the response to cytotoxic and therapeutic agents used in cancer treatment [24], reviewed in [23]. In agreement with this view, intestinal crypts lacking either pRb or E2F1-2-3 show increased DNA damage [4], perhaps due to a defect in the detoxification process caused by the absence of either regulator. A better understanding of the interactions between pRb, E2Fs and drug metabolizing enzymes could yield valuable insights to design more efficient cancer treatments as well as to help minimize adverse reactions to multiple pharmacological substances in genetically diverse patients.

## Materials and Methods

### Ethics statement

The protocols used for handling mice were approved by the Institutional Animal Care and Use Committees of the University of Pittsburgh (Protocol number 0805624) and the Ohio State University (Protocol number 2007A0239). All animal work was conducted according to relevant national and international guidelines. Mice were maintained in Animal Facilities according to University regulations and policies, without any dietary restrictions. Routine screens for mouse pathogens were negative.

### Production and maintanance of genetically altered mice

Generation of the conventional or conditionally inactivated alleles for RB, E2F1, E2F2 and E2F3 has been described [4]. Standard genetic crosses were performed to obtain mice lacking various genes in the intestinal epithelium (Rb^f/f^ or *RBKO*; E2F1-/-, E2F2 -/-, E2F3^f/f^ or *TKO*; Rb^f/f^, E2F1-/-, E2F2 -/-, E2F3^f/f^ or *QKO*). The genotype of each mice or embryo was determined by PCR analysis using DNA samples from murine tails and specific primers [4].

### Microarray analysis

The Affymetrix mouse whole genome chip consisting of 21,635 unique genes was used for microarray analysis. All microarray data is MIAME compliant and had been deposited at the Gene Expression Ommibus at the National Center for Biotechnology Information under accession number GSE16454, as described in our previous publication [4]. We focused on probes for genes belonging to Phase I (Cyp genes), Phase II (Gst, Ugt genes) and Phase III (Abc, Slc and Slco genes) of the detoxification pathway. The total number of genes which were 1.5 fold up or down within an individual detoxification group was evaluated. To be included in the analysis, an upregulated gene had to be present in all the three replicates of the experimental class (*RBKO, TKO* or *QKO*) and a downregulated gene had to be present in all the three replicates of the control class (control mice injected with β -naptoflavone).

### RNA obtention and transcriptional analysis

RNA was collected with either an RNAeasy kit (Qiagen, Valencia, CA, USA) or using the RNA-Bee method (Tel-Test, Friendswood, TX, USA). Synthesis of cDNA, RTPCR and real time PCR analysis have been described [8]. Each determination for real time analysis was carried out at least in triplicate and was considered valid only if the standard deviation was no higher than 0.3, and that the results shown are representative examples of at least two sets of treatments. Primers specific for each particular gene are detailed as follows:

Cyp1a1 (5′- GACCCTTACAAGTATGTCGT/5′-GGTATCCAGAGCCAGTAACCT), Cyp1b1 (5′-GCAACTTCAGCAACTTCGTTCTG/5′-CGGGTATCTGGTAAAGAGGATGAG), Cyp2c55 (5′-AATGATCTGGGGGTGATTTTCAG/5′- GCGATCCTCGATGCTCCTC), Cyp2d10 (5′-GGCAGAGATAGAGAAGGTAAAGGGG/5′-GACAGCATTGGTGTAGGGCATAC), Cyp3a11 (5′-GGAAAGCCGCCTGGATTCTAAG/5′-ATGCTGCCCTTGTTCTCCTTGC), Cyp3a25 (5′-CCGTTACTTGGCACCATTTT/5′-GTCTTTCATGCTGATGGGCT), Cyp4f13 (5′- CGACAGCAACTGCCAAGAGT/5′-CAGTGAGATAGTACAGGAGGTCC), Gstµ1 (5′-CCTGCCCACGTTTCTCTAG/5′-CGTGTAGCAAGGGCCTACTTG), Gst theta 1 (5′-TGTGTGAGAGTGTGGCTATCTTGC/5′TTATGATGAGGTCAGCAGGGGGAC), Mrp2 (5′-ACGGTCATCACTATCGCACACAGG/5′-TTGCTTGAGCCTTAGAGTTTGAGG), Rpl5 (5′-CCAAACGATTCCCTGGTAATGAC/5′-GACGATTCCACCTCTTCTTCTTCAC).

### Induction of the detoxification response in mouse embryos

A line containing a conventional inactivation of RB [18] was obtained from Jackson Labs. *RB +/-* females pregnant from *RB +/-* males were injected at E11.5 days with either PCN (pregnenolone-16 alpha-carbonitrile; 40 mg/kg) or with DMSO as a control. Embryonic livers were subsequently collected at E12.5 days and processed for RNA extraction. To allow *RB -/-* embryos to survive after E13.5, we used *RB* loxP/loxP conditional knockout and Mox2 +/cre transgenic mice to supply pRb-deficient embryos with wild-type placentas, as described in [25]. Pregnant females were injected with PCN one day prior to sacrifice, and the livers or intestines of E18.5 day embryos were dissected and processed to obtain either RNA or proteins using standard methods.

### Tissue manipulation and protein analysis

Obtention of intestinal fractions enriched for villi and/or crypts has been described [26]. Protein extraction with RIPA buffer (PBS 1x, NP-40 1%, Sodium deoxycholate 0.5%, SDS 0.1%) in the presence of protease inhibitors and western blot analysis were performed using standard techniques. Cyp3a L-14 (Santa Cruz Biotechnology Inc.), an antibody detecting several Cyp3A submembers, was used in these experiments.

## Supporting Information

Table S1 Summary of detoxification genes downregulated in villi and crypts from *RBKO* mice.
(DOC)

Table S2 Summary of detoxification genes downregulated in villi and crypts from *TKO* mice.
(DOC)

Table S3 Summary of detoxification genes downregulated in villi and crypts from *QKO* mice.
(DOC)
