# Supplementary figures and images for: The Retinoblastoma Tumor Suppressor Regulates a Xenobiotic Detoxification Pathway
Source: PLoS One. 2011 Oct 12;6(10):e26019. doi: 10.1371/journal.pone.0026019 (PMC3192141; doi:10.1371/journal.pone.0026019)

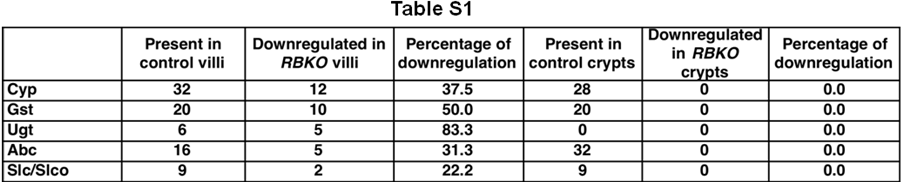

Supplement: Table S1 — Summary of detoxification genes downregulated in villi and crypts from RBKO mice. (DOC) [file pone.0026019.s001.doc]

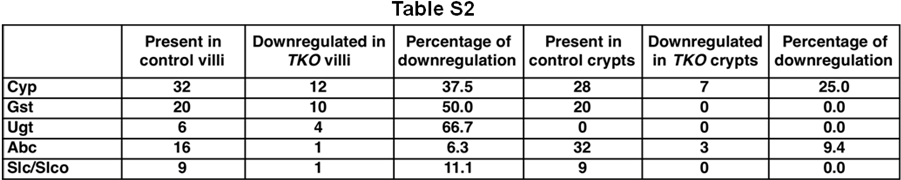

Supplement: Table S2 — Summary of detoxification genes downregulated in villi and crypts from TKO mice. (DOC) [file pone.0026019.s002.doc]

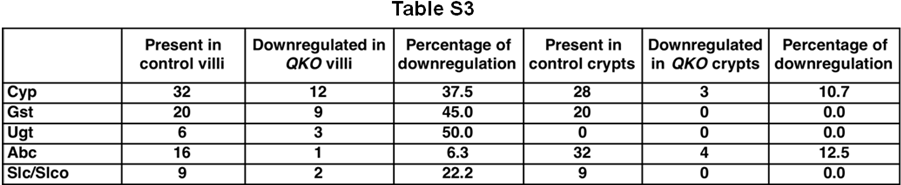

Supplement: Table S3 — Summary of detoxification genes downregulated in villi and crypts from QKO mice. (DOC) [file pone.0026019.s003.doc]
